# Editorial: Personalized surgery of the face

**DOI:** 10.3389/fsurg.2024.1517356

**Published:** 2024-11-13

**Authors:** Sergio Olate, Marcio de Moraes

**Affiliations:** ^1^ Department of Oral and Maxillofacial Surgery, Faculty of Dentistry, Universidad de La Frontera, Temuco, Chile; ^2^ Center for Research in Morphology and Surgery (CEMyQ), Faculty of Medicine, Universidad de La Frontera, Temuco, Chile; ^3^ Division of Oral and Maxillofacial Surgery, Piracicaba Dental School, Campinas State University, Piracicaba, Brazil

**Keywords:** face, surgery, personalized medicine, facial plastic & reconstructive surgery, maxillofacial surgery, technology

**Editorial on the Research Topic**
Personalized surgery of the face

The human face presents individual conditions in structural morphology and function related to conditions of the overall health of individuals; the relationship between the adequate facial form and functional conditions are closely linked. In this manner, the presence of certain craniomaxillofacial pathologies or conditions that were previously considered separate entities, are currently accepted as part of the same problem, as a result of comprehensive facial analysis based on contemporary methods that include artificial intelligence, three-dimensional printing, virtual planning, robotic surgery and the application of biomaterials among others.

Therapeutic approaches have varied over time in both techniques and technologies, rendering personalization an important aspect in defining planning, treatment, and prognosis. This special issue presents important information on new technologies and treatment options in different facial conditions. The article by Knoedler et al. realize a detailed analysis regarding applications of artificial intelligence in cases of vascularized facial transplantation, including certain algorithms that can be applied for planning, simulating the results and outlining the surgery. They performed a detailed analysis of AI applied to the prediction of rejection and the possibility of malignant transformation in the treatments carried out.

Ureel et al. present an analysis for the use of alloplastic nose reconstruction using 3D technology; the case presented includes an 11-year-old patient treated with implants integrated in the peripheral area of the nose, without injury to the teeth for implant placement; a bar is connected to the implants and allows anchoring of a prosthesis that simulates the nasal shape in its anterior and basal area with good aesthetic results; the management option is discussed to be used in child and adolescent patients.

The study by Han et al. is an interesting analysis of facial contour basal osteotomy using specific printed guides for the mandibular angle area; the sequence of 11 patients analyzes planning considering the position of the inferior alveolar nerve and location of the surgical guide, noting symmetry in the digital planning; although there was an explicit mention of lipofilling in the area, its real impact could not be confirmed in the final aesthetic assessment.

The concept of hydroxyapatite-based facial implants was introduced by Kauke-Navarro et al.; the authors performed a systematic review including 12 studies with 74 patients treated with this system involving implants in the orbital-maxillary area, frontal area and mandible area; despite limitations in the studies such as randomization, comparison with other techniques and long-term follow-ups, they present favorable results in the analyses carried out.

Personalization and precision of surgical intervention was analyzed by Han et al., who present research on robotic assistance and the learning curve for mandibular angle osteotomy; to achieve this aim they use an animal model with 30 rabbits where they observed that the use of robotic assistance reduces the time to perform the osteotomy, increases precision, decreases the risk of complications and improves surgery results; this evidence presents the initial results for the use of robot-assisted teaching of surgical techniques in the craniomaxillofacial area.

The last article in this special issue was presented by Pulino et al., who emphasize the importance of using customized implants in cases of reconstruction due to sequelae of facial trauma. The authors perform an analysis of the use of orbital implants designed and printed in titanium with specific application, highlighting the predictability and postoperative stability in cases of severe sequelae; a specific analysis on the different materials is presented in this study.

The articles in this special issue analyze the current technology for the planning and technical execution of facial surgeries, including reconstructive and aesthetic aspects. However, there are aspects that require in-depth study, such as the conditions of facial development and aging and their impact on the selection of the best surgical technique, as well as the psycho-emotional and cultural conditions of patients who are candidates for reconstructive and aesthetic surgery, understanding the parameters of modern society.

Throughout history, art has seen facial morphology in sculptures and paintings, including proportional and harmonious aspects of the face to identify individual characteristics; modern day technology allows us to better understand facial normalcy and include innovative options, in order to attain highly predictable results and reduce complications.

Undoubtedly personalized facial surgery is an important challenge for scientists and surgeons who intervene at different levels of the face, making its comprehensive study increasingly relevant for the appropriate indication of different techniques available to specialists today.

